# The Role of Histamine in the Retina: Studies on the Hdc Knockout Mouse

**DOI:** 10.1371/journal.pone.0116025

**Published:** 2014-12-29

**Authors:** Ursula Greferath, Kirstan A. Vessey, Andrew I. Jobling, Samuel A. Mills, Bang V. Bui, Zheng He, Nupur Nag, Hiroshi Ohtsu, Erica L. Fletcher

**Affiliations:** 1 Department of Anatomy and Neuroscience, The University of Melbourne, Parkville, Victoria, Australia; 2 Department of Optometry and Vision Sciences, The University of Melbourne, Parkville, Victoria, Australia; 3 Department of Engineering, Medical School of Tohoku University, Sendai, Japan; Universidade Federal do ABC, Brazil

## Abstract

The role of histamine in the retina is not well understood, despite it regulating a number of functions within the brain, including sleep, feeding, energy balance, and anxiety. In this study we characterized the structure and function of the retina in mice that lacked expression of the rate limiting enzyme in the formation of histamine, histidine decarboxylase (Hdc^−/−^ mouse). Using laser capture microdissection, *Hdc* mRNA expression was assessed in the inner and outer nuclear layers of adult C57Bl6J wildtype (WT) and Hdc^−/−^-retinae. In adult WT and Hdc^−/−^-mice, retinal fundi were imaged, retinal structure was assessed using immunocytochemistry and function was probed by electroretinography. Blood flow velocity was assessed by quantifying temporal changes in the dynamic fluorescein angiography in arterioles and venules. In WT retinae, *Hdc* gene expression was detected in the outer nuclear layer, but not the inner nuclear layer, while the lack of *Hdc* expression was confirmed in the Hdc^−/−^ retina. Preliminary examination of the fundus and retinal structure of the widely used Hdc^−/−^mouse strain revealed discrete lesions across the retina that corresponded to areas of photoreceptor abnormality reminiscent of the rd8 (*Crb1*) mutation. This was confirmed after genotyping and the strain designated Hdc^rd8/rd8^. In order to determine the effect of the lack of Hdc-alone on the retina, Hdc^−/−^ mice free of the Crb1 mutation were bred. Retinal fundi appeared normal in these animals and there was no difference in retinal structure, macrogliosis, nor any change in microglial characteristics in Hdc^−/−^ compared to wildtype retinae. In addition, retinal function and retinal blood flow dynamics showed no alterations in the Hdc^−/−^ retina. Overall, these results suggest that histamine plays little role in modulating retinal structure and function.

## Introduction

It is well recognized that the principal neurotransmitters of the mammalian retina are the amino acid neurotransmitters glutamate, gamma-aminobutyric acid (GABA) and glycine [1]. However, there is co-localization of amino acid neurotransmitters with a range of other neurotransmitters and neuromodulators in the inner retina, within amacrine cells [2]–[6]. Histamine is a well characterized neuromodulator of the Central Nervous System (CNS) [7], however, its role in modulating retinal circuits and vision is less well understood.

Histamine is produced within mast cells and neurons in the CNS [7]. Histamine decarboxylase (Hdc) is the rate limiting enzyme in the formation of histamine, catalyzing the synthesis of histamine from the amino acid L-histidine [8]. The actions of histamine are mediated by four different histamine receptors (H_1_R-H_4_R) which induce neural effects via G-protein coupled signaling mechanisms [7]. Within the CNS, the tuberomammillary nucleus of the hypothalamus is the principal site for neuronal synthesis of histamine. Histaminergic neurons project from the tuberomammillary nucleus to a widespread number of regions throughout the cortex [9]. These histaminergic projections are thought to be important in regulating sleep-wakefulness, feeding and energy balance. Anomalies in its signaling has been associated with a range of disorders including anxiety, depression, narcolepsy and Tourette's syndrome [7]. More recently, a single mutation in the *Hdc* gene (W317X) has been associated with Tourette's syndrome in one family [10].

In the retina, no histamine forming cells have been identified to date. Rather, sparse retinopetal axons arising from the tuberomammillary nucleus extend across the inner plexiform layer eliciting responses in a range of inner retinal neurons [11]–[13]. Histamine receptors, H_1_R, H_2_R, H_3_R have been localized to subsets of inner retinal neurons in rodent and primate retinae [13], [14]. In particular, circuits important in mediating scotopic vision, may be altered by histamine release. Notably, dopaminergic amacrine cells express H_1_R, and display altered intracellular calcium responses, when activated by H_1_R agonists [15]. In addition, histamine has been shown to reduce the sensitivity of ON ganglion cells to light, especially under dark adapted conditions [16]. Histamine may also be important in the regulation of ocular blood flow, via alterations in vessel caliber [17], [18]. Recently, histamine signaling has been implicated in the pathology underlying age related macular degeneration (AMD), with H_4_R expression increased in the eyes of AMD patients [19]. As this receptor is primarily expressed by macrophages of the eye and the use of antagonists to H_4_R may reduce choroidal neovascularisation [19].

A great deal has been learned about the role of histamine in the CNS from examining changes in behavior and function of Hdc^−/−^mice. For example, Hdc^−/−^mice display aberrant sleep-wake patterns, reflecting histamine's role in sleep and wakefulness [20]. Furthermore, the association between neural dysfunction and the lack of *Hdc* gene expression in humans has been confirmed in studies using the Hdc^−/−^ mouse with histamine modulating dopamine signaling within the basal ganglia [10]. To date there have been no reports of retinal changes or functional anomalies in the Hdc^−/−^ mouse. Based on the expression and distribution of histamine receptors within the retina [13], [14], and the functional evidence that some retinal neurons are modulated by histamine [12], it is likely that the Hdc^−/−^ mouse has a retinal phenotype.

The aim of this study was to evaluate changes in retinal structure and function in Hdc^−/−^ mice. Based on previous reports that histamine elicits a range of functional changes, especially in the scotopic retinal circuits [12], we predicted that retinal function in Hdc^−/−^ mice would be affected, especially that of amacrine cells. Unexpectedly, our results showed that in the absence of histamine, retinal structure and function was unchanged in the Hdc^−/−^ mice compared to wildtype controls. Our results suggest that histamine plays a minor, if any, role in modulating the major rod and cone mediated circuits. However, caution is needed when interpreting results from experiments using Hdc^−/−^ mice, since our work revealed that the commonly available strain carries a background mutation in Crb1 that affects retinal structure and photoreceptor viability.

## Materials and Methods

### Animals

All procedures concerning animals were approved by the University of Melbourne Animal Experimentation Ethics Committee (AEC#1112259) and were conducted in accordance with guidelines set by the National Health and Medical Research Council. All experiments adhered to the ARVO statement for the use of animals in ophthalmic and vision research.

Adult Hdc^−/−^ mice were originally obtained from Prof H Ohtsu (Tohoku University, Sendai Japan) and are engineered as previously described [21]. Briefly, Hdc^−/−^ mice were originally created by replacing intron 5 to exon 9 of the murine *Hdc* gene with an inverted PGK promoter driven neomycin phosphotransferase gene. This lead to targeted deletion of exon 8, which contains the coding sequence for the putative binding site for pyridoxal 5′-phosphate, the coenzyme of Hdc protein. Control, wildtype C57Bl6 mice (WT), and Hdc^−/−^ mice were housed at the Animal Research Facility of the Faculty of Medicine and Health Sciences, University of Melbourne, Victoria, Australia under standard conditions with food and water provided *ad libitum* in a 12 hour light, 12 hour dark cycle. Ambient light in the animal house was measured using a photometer and was found to be between 9 lux and 129 Lux depending on the shelf used to house animals. All mice evaluated in this study were aged 2–4 months.

Hdc^−/−^ mice are able to obtain histamine via their diet. Thus, in order to study the effects of histamine depletion on retinal structure and function, it was necessary to raise animals on a histamine-free diet. All experimental mice were kept on a histamine-free diet (Standard AIN93M Rodent Diet from Speciality Feeds, Glen Forrest, WA, Australia) for at least for 10 days before experiments. As previously described [22], neural tissue is depleted of histamine in 8 days leading to a range of CNS anomalies [10], [20], [22].

During the course of the study, a mutation within the *Crb1* gene (designated rd8/rd8) was identified in the background strain. This mutation has been identified in several other mouse lines used in ophthalmic/vision research [23]. In order to determine the effect of the lack of histamine on retinal structure and function, we bred out the rd8/rd8 mutation by backcrossing mice more than ten generations onto the C57Bl6J line. Throughout the paper we use the term Hdc^rd8/rd8^ to denote our studies using the original strain containing the *Crb1* mutation, while Hdc^−/−^ is used to denote the animals lacking Hdc, yet verified to be free of the *Crb1* mutation.

### Genotyping

Small tail samples were collected from wildtype, Hdc^rd8/rd8^ and Hdc^−/−^ mice after weaning and genomic DNA extracted. Standard PCR based genotyping (MyTaq, Bioline London, UK) using mouse-specific primers was performed to amplify a 147bp fragment from WT animals (forward, 5-AGT GAG GGA CTG TGG CTC CAC GTC GAT GCT-3, reverse 5-TAC AGT CAA AGT GTA CCA TCA TCC ACT TGG-3) while primers within the neo^r^gene were used to amplify a 244bp fragment from the Hdc^rd8/rd8^ and Hdc^−/−^ animals (5-AAA CAT CGC ATC GAG CGA GCA CGT AC T CGG-3 and 5-ATG TCC TGA TAG CGG TCC GCC ACA CCC AGC -3). The amplified products were purified (Qiaquick, Qiagen, Valencia CA) and sequenced (Australian Genome Research Facility, Melbourne, Australia) to confirm the identity of the fragment. To ensure the re-derived Hdc^−/−^ mice lacked the Crb1 mutation, PCR was performed on the *Crb1* gene using allele-specific PCR, as specified in [24]. As with all amplified products, these samples were subsequently sequenced (Australian Genome Research Facility) to confirm the presence of the wildtype *Crb1* sequence.

### Retinal fundus photography

Three month old WT, Hdc^rd8/rd8^ and Hdc^−/−^ mice were anaesthetized using a mixture of ketamine (67 mg/kg) and xylazine (13 mg/kg) and the ocular surface further anaesthetized with topical proxymetacaine (Alcaine, 0.5% Alcon Laboratories, Frenchs Forest, NSW, Australia). Following dilation of the pupil with 0.5% tropicamide (Mydracyl, Alcon Laboratories) and 10% Phenylephrine hydrochloride (Minims Eye Drops, Bausch & Lomb, Macquarie Park, NSW, Australia), mice were placed in a special holder and the retinal fundi imaged with a Micron III fundus camera (Phoenix Research Laboratories, Pleasanton, CA) as previously described [25]. Fundus images were collected and processed using the Micron III specialty software (StreamPix 5.0 NorPix Inc., Quebec, Canada).

### Immunohistochemistry

There are many studies that have demonstrated CNS anomalies in Hdc^−/−^ mice [10], [20], [26]. We first verified that Hdc^−/−^ mice lack histamine using antisera specific to histamine in brain sections containing the tuberomammillary nucleus as described previously (Greferath et al 2009). Briefly, Hdc^−/−^ and WT mice were deeply anesthetized by intraperitoneal injection of a mixture of ketamine and xylazine (in an overdose to above) and transcardially perfused with ice-cold, freshly prepared 4% 1-ethyl-3-(3diethylaminopropyl)-carbodiimide in 0.1 M phosphate buffer, pH 7.4 (PB). Brains were dissected and postfixed overnight in the same fixative. Brains were cryoprotected and snap frozen in Tissue Tek Optimal Cutting Temperature (OCT, Sakura Finatek Inc., Torrance, CA). 20 µm cross sections were taken from the region of the tuberomammillary nucleus on a cryostat and collected onto slides coated with polysine (Menzel-Glaser, Germany). The sections were stored at −70°C or stained directly for immunohistochemistry. Sections were incubated overnight in anti-histamine antisera in a solution containing 3% Normal Goat Serum (NGS), 1% BSA, 0.01% Triton-X-100 in PB. Sections were then washed in PB and incubated for 1.5 hours in goat anti-rabbit conjugated to AlexaFluor 594, diluted 1∶500 in 3% NGS, 1% BSA, 0.01% Triton-X-100 in PB. A nuclear dye, 4′,6-diamidino-2-phenylindole (DAPI; diluted 1∶300; Life Sciences) was also added to the tissue sections and after final rinsing sections were coverslipped using fluorescent mounting media (DAKO, Carpinteria, CA). Brain sections were viewed and imaged using a Zeiss LSM-5 confocal microscope (Zeiss, Oberkochen, Germany).

Histamine immunolabeled cells were detected in the tuberomammillary nucleus of the hypothalamus of WT mice (S1A Fig.). Histamine immunolabeling was absent from the tuberomammillary nucleus of Hdc^−/−^-mice raised raised for ten days on a histamine-free diet (S1B Fig.).

In order to examine the integrity of the WT and Hdc^−/−^-retina, indirect immunofluorescence was performed as previously described [25], [27]. Briefly, following death, eyes were removed, a small incision made into the eyecup and the eye subsequently dissected in 4% paraformaldehyde (PFA) in PB. The anterior eyecup and lens were removed and the posterior eyecups containing the retinae were further fixed for 30 min in the same fixative. Posterior eyecups were cryoprotected and finally equilibrated in 30% sucrose overnight and snap frozen in Tissue Tek. Eyes were sectioned at 12–16 µm on a cryostat and sections collected onto polysine coated slides.

For immunocytochemistry, vertical sections and wholemounts were incubated overnight (sections) or for 3 nights (wholemounts) in primary antisera (see Table 1) diluted in a solution containing 3% NGS, 1% BSA, 0.01% Triton-X-100 in PB. These antisera are known markers of various cells types in the retina [28], [29], including cone photoreceptors (peanut agglutin, PNA), rod bipolar cells (Protein kinase Cα, PKCα), amacrine and ganglion cells (Calretinin), dopaminergic amacrine cells (tyrosine hydroxylase, TH), microglia (Ionized calcium binding adaptor molecule 1, IbA1), Müller cells (glutamine synthetase, GS), astrocytes (glial fibrillary acidic protein, GFAP). Sections were then washed in PB and incubated for 1.5 hours (sections) or overnight (wholemounts) in secondary antisera: goat anti-mouse, or goat anti-rabbit conjugated to fluorescent dyes (AlexaFluor 488, AlexaFluor 594, or AlexaFluor 643; Life Sciences, VIC, Australia) diluted 1∶500 in 3% NGS, 1% BSA, 0.01% Triton-X-100 in PB including DAPI and mounted with mounting media.

**Table 1:** List of antisera used in this study.

Name	Immunogen and cellular label	Dilution	Source
Rabbit Anti-Histamine	Histamine conjugated to keyhole limpet hemocyanin with 1-ethyl-3(3-dimethylaminopropyl)-carbodiimide)	1∶500	Cat# AB5885; Millipore, Merck, VIC, Australia [13]
Rabbit Anti-Crb1	36 amino acid peptide targeting the C-terminus of human Crb1.	1∶250	J Winjholds, Amsterdam [47].
Mouse anti- Calretinin	Recombinant human calretinin - 22k. Labels amacrine and ganglion cells	1∶1000	Cat# 63B; Swant, Bellinzona, Switzerland
Fluorescein labelled Peanut Agglutinin (PNA) from *Arachis hypogaea* (peanuts)	Labels cone photoreceptors.	1∶250	Cat# FL-1071; Vector Laboratories, Burlingame, CA
Mouse Anti-Protein Kinase Ca monoclonal (MC5)	Peptide Amino acids 296-317 of PKC. Labels rod bipolar cells.	1∶400	Cat# P5704; Sigma-Aldrich, NSW, Australia [48], [49]
Mouse Anti-Tyrosine hydroxylase	Tyrosine hydroxylase purified from PC12 cells. Labels tyrosine hydroxylase immunoreactive amacrine cells.	1∶1000	Chemicon International (Temecula, CA, USA), #MAB318, Lot#22061050 mouse monoclonal [50]
Mouse anti-glutamine Synthetase (GS), monoclonal (GS-6)	Glutamine synthetase purified from sheep brain. Labels Müller cells	1∶1000	Cat# MAB302; Millipore, Merck, VIC, Australia [30]
Rabbit anti-glial fibrillary acid protein (GFAP), polyclonal	Bovine spinal cord GFAP. Labels retinal astrocytes and gliotic Müller cells.	1∶20,000	Cat# Z0334;Dako,Carpinteria, CA, USA[30]
Rabbit anti-Ionized calcium binding adaptor molecule 1 (IbA1), polyclonal	Synthetic peptide corresponding to the C-terminus of IbA1: PTGPPAKKAISELP. Labels microglia.	1∶1500	Cat# 019-19741; Wako Pure Chemical Industries, Richmond, VA, USA [30]

Retinae were viewed and imaged using a Zeiss LSM-5 confocal microscope (Zeiss, Oberkochen, Germany). Air (X20) and oil (X40) objectives were used to view labelled sections. Images were captured at a resolution of 1024 by 1024 pixels using Zeiss LSM image browser software and an appropriate fluorescence filter (Alexa TM 594/CY3: excitation 568 nm, emission filter 605/32; Alexa TM 488/FITC: excitation 488 nm, emission filter 522/32). Red and green fluorescence was scanned separately and adjusted for black levels, brightness and contrast with Adobe Photoshop CS4 (Adobe Systems, San Jose, CA).

In order to quantify the density of specific retinal cell types, vertical sections of wildtype (two-four sections per animal, N = 7 mice) and Hdc^−/−^ retinae (two-four sections per animal N = 9 mice) were imaged at 40× oil and the number of PKCa, TH, calretinin positive cells in the ganglion cell layer quantified by millimeter of retinal section length. In addition we quantified the number of TH immunoreactive cells in the central retina of flatmounted wildtype (N = 7 mice) and Hdc^−/−^ (N = 5) retinae. The total thickness of the central retina and inner plexiform layer was also quantified by measuring the distance from the Inner limiting membrane to the outer limiting membrane of wildtype (N = 7 mice) and Hdc^−/−^ mice (N = 9 mice). Differences in the density of cells or retinal thickness was assessed using Graphpad Prism 6.0 using an unpaired t-test. An alpha of 0.05 was adopted for statistical purposes.

### Laser-capture microdissection and RT-PCR

Cryostat sections were prepared as above and washed in 50% ethanol and then dehydrated in 100% ethanol. Samples of either the outer nuclear layer (ONL) or the inner nuclear layer (INL) were microdissected with a Palm Laser Dissector System (Zeiss) and collected into 50 µl of lysis solution for purification of total RNA from fixed tissue sections (buffer PKD, RNeasy FFPE kit, Qiagen). Total RNA was extracted and primer specific RT-PCR was performed using the One-Step RT-PCR (Qiagen). Primers to Rhodopsin (*Rho*) and *Gad-67*, were used to confirm the purity of the ONL versus INL samples, respectively (Table 2). In order to detect two different portions of histamine decarboxylase (Hdc), two different forward primers in combination with one reverse primer specific to *Hdc* were designed and used resulting in two different sized products (Table 2). Two different regions of Hdc were amplified to verify more accurately expression in the different retinal regions. The One step RT-PCR conditions were 50°C for 30 min, 95°C for 15 min, 95°C for 4 minutes (1 cycle each), 94°C for 30 sec, 55°C for 30 sec, 72°C for 1 min (46 cycles), 72°C for 10 minutes. Amplified products were separated by electrophoresis on a 1.5% agarose gel, extracted (Qiaquick, Qiagen) and sequenced (Australian Genome Research Facility) to confirm the identity of the products.

**Table 2:** Primer sequences used in this study.

Primer name	Primer sequence
Gad-67 forward	5-ATGCAACCGCAGGCACGACT-3
Gad-67 reverse	5-ACCACCCCAGGCAGCATCCA-3
Hdc- forward 110	5-GGTGCCTGTGTTTGTCTGTGC-3
Hdc- forward 275	5-CTACACCTCTGATCAGGCTCAC-3
Hdc- reverse	5-TCCCTCACTGGCACAGATGGG-3
Rho- forward	5-AGCAGCAGGAGTCAGCCACC-3
Rho - reverse	5-CCGAAGTTGGAGCCCTGGTG-3

### Morphological analysis on paraffin sections

For paraffin sectioning mice were killed by cervical dislocation their eyes dissected as above, and placed in a fixative containing 4% PFA, 3% sucrose, 5% acetic acid in 60% ethanol and fixed for 1–3 days. Tissues were then dehydrated in a series of ethanol and histolene (Grale Scientific Ringwood, Australia) and embedded in paraffin. Sections were cut (5 µm), dewaxed and stained with Haematoxylin-Eosin. They were then washed in water, dehydrated in ethanol series, cleared in Histolene and mounted and in Safety Mount (Fronine, Riverstone, Australia).

### Retinal function: the electroretinogram

Retinal function was measured by electroretinography using a twin flash paradigm to separate rod from cone mediated responses, as previously described [25], [30]. Briefly, mice were dark adapted overnight, anaesthetized with a mixture of ketamine and xylazine (as above), and the corneas further anaesthetized with topical 0.5% proxymetacaine and pupils dilated with 0.5% tropicamide (Mydriacyl, Alcon Laboratories). Two electrodes were placed on the animal: a custom made silver/silver chloride electrode placed on the centre of the cornea and a reference electrode placed in the animal's mouth. Rod and cone responses were isolated using a twin flash paradigm, whereby two bright flashes of 2.1 log cd.s/m^2^ intensity were generated by a Nikon photography flash (Nikon SB900, NSW Australia) delivered through a Ganzfeld bowl and presented 0.8 s apart. The first flash elicits a mixed (rod and cone) response, whereas the second flash elicits responses from neurons forming the cone pathway [31], [32]. Digital subtraction of these two responses derives function in the rod pathway. Coordination of ERG stimulation and recording of electrical responses was completed using Scope v3.6.9 software and the responses were filtered for 60 Hz noise, amplified and digitized at 10 kHz over a 250 ms epoch (gain×5000; −3 dB at 1 Hz and 1 kHz, ADInstruments, NSW, Australia).

In order to examine the changes in function of individual classes of neurons, we performed a component analysis on the raw data as previously described [28], [33]. Rod photoreceptor responses (rod a-wave) were analysed using a modified PIII model and described in terms of the amplitude of the PIII response (PIII Rmax in µV) and its sensitivity, (S in m^2^cd^−1^s^−3^) [28], [31]. The rod post-photoreceptoral function (rod b-wave) was isolated by subtraction of the rod PIII from the raw rod waveform and then fitted using an inverted gamma function to generate the rod PII, to return the amplitude (rod PII Rmax in µV) and time to peak (implicit time in ms). Oscillatory Potentials, reflecting function of inner retinal neurons, especially amacrine cells, were extracted by removing the fitted PII response from the raw waveform and the amplitude (µV) and implicit time (ms) of OP2, OP3 and OP4 were analyzed.

Mice are known to be rod dominated, having only a small number of cones. Thus, it is not possible to measure a cone a-wave. However, the cone post-receptoral response (cone b-wave) could be assessed and the cone PII was analyzed by fitting an inverted gamma function to the raw cone waveform. From the cone PII fit, the amplitude of the cone PII response (cone PII Rmax in µV), and the time to peak (Implicit time in ms) were determined. The cone pathway driven, oscillatory potentials were extracted by removing the fitted cone PII response from the raw waveform and the amplitude (µV) and implicit time (ms) of OP1, OP2 and OP3 were analyzed.

ERG data were modelled and analyzed using Excel (Microsoft Office Excel, Microsoft, Redmond, WA), and statistical analysis was performed using GraphPad Prism 5 (GraphPad Software, San Diego, CA). Data are presented as mean ± SEM and were analyzed using a Student's t-test, where a difference was considered significant if p<0.05.

### Measurement of blood flow velocity: video fluorescein angiography

In order to assess whether histamine influenced retinal blood flow, fluorescein angiography was performed, captured in real time and analysed as previously described [34]. Briefly 5 Hdc^−/−^ and wildtype mice were anaesthetized with a combination of ketamine and xylazine, as described above. Following animal preparation as per fundus photography, a bolus of contrast media, sodium fluorescein (0.1%, 10 µl/kg), was infused at 0.3 ml/min for 4 seconds using a syringe pump (11 Plus, Harvard Apparatus, MA, USA) via a femoral vein cannula (polyethylene tubing inner diameter 0.2 mm, outer diameter 0.4 mm, Microtube Extrusions, NSW, Australia). Images were taken from the start of fluorescein infusion with the Micron III camera (Phoenix Research Labs) with 435–469 nm excitation and 520–530 nm barrier filters at 30 frames/sec using Streampix (NorPix Inc., Quebec, Canada) for 30 seconds. Data were saved as tiff image stacks for offline analysis.

Images stacks were registered via x/y translation using custom written MATLAB scripts (R2013a, The MathWorks Inc., Massachusetts, USA) via cross-correlation to a reference image at peak fluorescence (∼15 seconds from onset of infusion). Following registration, masks were manually created to demarcate the major arterioles and venules for analyses. Capillaries were isolated by using the negative of these masks combined. Pixel intensity over time was measured for each vessel type, and from these response profiles the time to half peak, the gradient of the rise, and the time to half fall were measured and compared between WT and Hdc^−/−^ mice.

## Results

**Figure 1:**
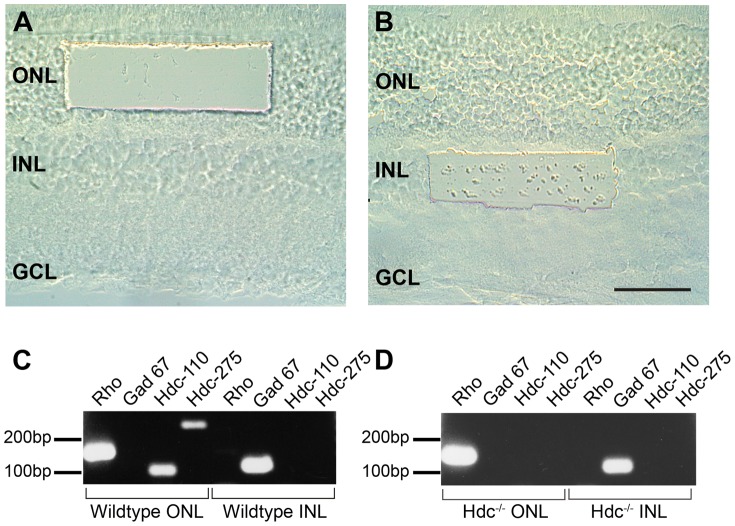
Expression of *Hdc* in the mammalian retina. Different regions of the WT and Hdc^−/−^ retina were isolated using a laser dissecting microscope. (A) shows the region of retina dissected from ONL and (B) is region from INL. Total RNA was prepared from the ONL and INL slices and cDNA fragments amplified using primers specific to Rhodopsin (*Rho*), Glutamic acid dehydrogenase (*GAD*) 67, and the *Hdc* gene, of which two different sized fragments were amplified (*Hdc* - 110 and 275 bp). Amplified fragments isolated from wildtype (C) or Hdc^−/−^ (D) were separated on agarose gels as indicated in (C) and (D). GCL, ganglion cell layer; INL, inner nuclear layer; ONL, outer nuclear layer. Scale bar  = 50 µm.

Many studies have documented CNS anomalies in Hdc^−/−^ mice [10], [20], [26]. The central aim of this study was to evaluate the retinal manifestations of the absence of histamine by examining the Hdc^−/−^ mouse retina. We first assessed whether the rate limiting enzyme for histamine formation histidine decarboxylase (Hdc) was expressed in the retina and whether there was a specific localization of Hdc expression. RT-PCR was performed on laser dissected retinal samples, where the dissected outer retina (outer nuclear layer, ONL) contained the cell bodies of the photoreceptors (Fig. 1A), while the region dissected from the inner retina was restricted to the cells of the inner nuclear layer (INL, Fig. 1B). Analysis of samples from C57Bl6J (WT) mice are shown in Fig. 1C, while Hdc^−/−^ samples are shown in Fig. 1D. In both WT and Hdc^−/−^ retinae, ONL samples were positive for the rod photopigment, rhodopsin (*Rho*), but not for the amacrine cell marker *GAD-67*, while cells isolated from the INL were positive for *GAD-67*, but not *Rho*. These results validate the specificity of the microdissection procedure. Regarding *Hdc* expression in the WT retina, the two different sized fragments of the *Hdc* gene were amplified in samples taken from the outer retina, whereas samples from the inner retina did not show any amplified product (Fig. 1C). In samples isolated from the Hdc^−/−^, no amplified gene product was observed in either the outer or inner retina (Fig. 1D). These findings indicate that photoreceptors, but not inner retinal neurons, most likely express *Hdc* in the WT mouse retina.

### Analysis of retinal structure in the Hdc^rd8/rd8^ mice strain

**Figure 2:**
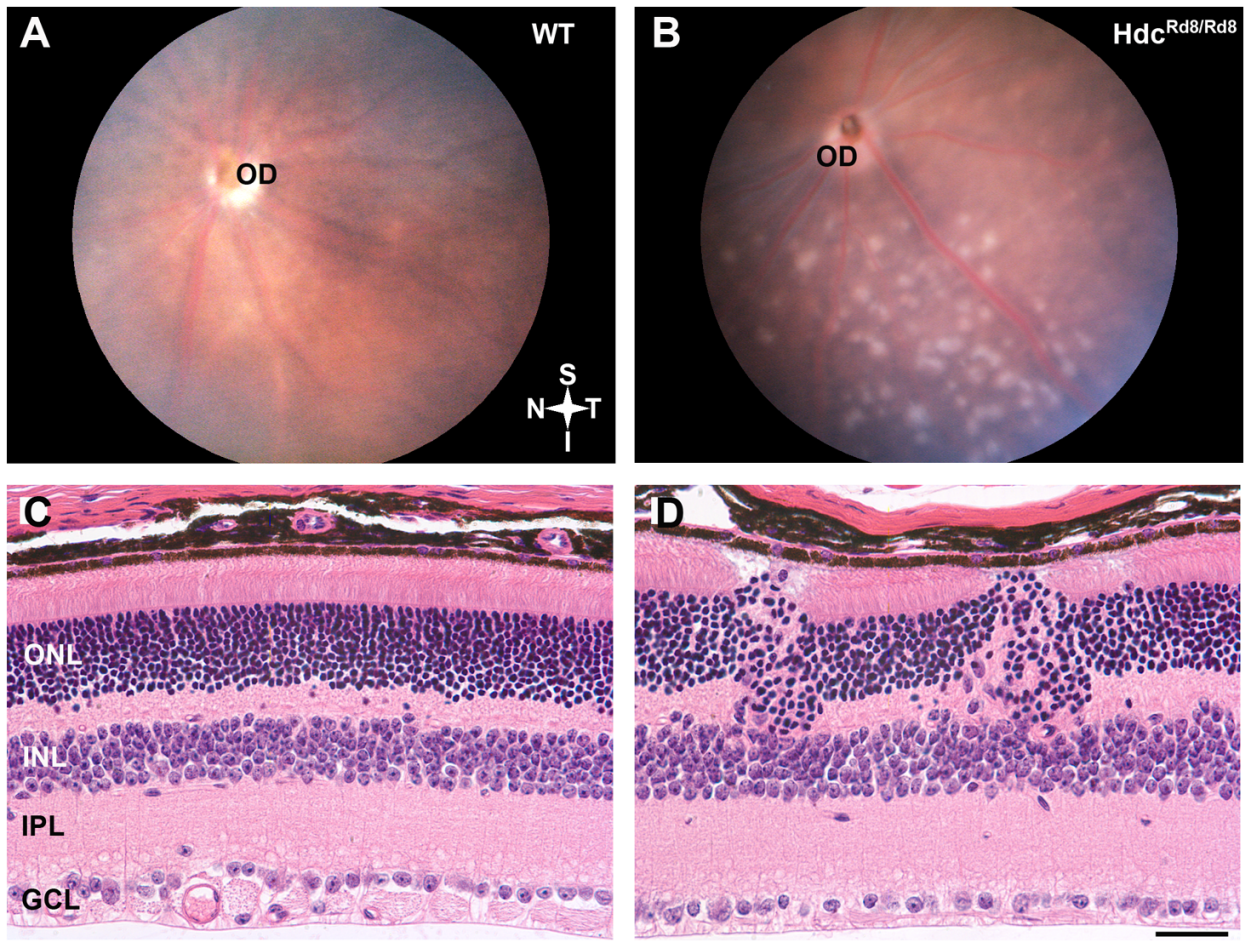
Hdc^rd8/rd8^ mice exhibit defects in the outer retina. Retinal fundus images of a three month of WT (A) and Hdc^rd8/rd8^mouse retina (B). Discrete white lesions were observed in the inferior retina in Hdc^rd8/rd8^ mice. Paraffin sections are shown of retinae from wild type (C) and Hdc^rd8/rd8^ mice (D). Disruptions in outer retina were observed in the Hdc^rd8/rd8^ mice. Abbreviations: OD-optic disc; ONL-outer nuclear layer; INL-inner nuclear layer; IPL-inner plexiform layer; GCL-Ganglion cell layer; Scale bars  = 50 µm.

In order to determine whether the loss of Hdc affected the retina, fundus appearance and retinal structure were investigated. As can be observed in Fig. 2, the fundus of age-matched WT mice appeared normal (Fig. 2A), while the Hdc^rd8/rd8^animals exhibited the accumulation of white spots in the inferior retina (Fig. 2B). When retinal structure was compared to WT mice (Fig. 2C), the outer retina of the Hdc^rd8/rd8^ strain was severely affected (Fig. 2D). Clumps or whorls of cells were seen in the outer nuclear layer (ONL), often giving the appearance of a thickened retina. There were a number of rosettes in the ONL, where the normal polarity of photoreceptors was disrupted. Notably, photoreceptor outer segments faced towards one-another, rather than sitting within the microvilli of the underlying Retinal Pigment Epithelium (RPE) (not shown). This fundus appearance and aberrant retinal structure is similar to that described for the retinal degeneration-8 mouse model (rd8) in which the gene *Crb1* is altered. As recent reports have documented the presence of this mutation in the background of numerous mouse strains used in vision research [23], the Hdc^rd8/rd8^strain was investigated for the *Crb1* mutation. Genotyping of these animals confirmed the presence of the *Crb1* mutation in addition to the targeted deletion of *Hdc* (strain renamed Hdc^rd8/rd8^).

### Analysis of retinal structure in Hdc^−/−^ mice free of Crb1

In order to examine the potential role that histamine has in retinal signaling independent of the rd8 mutation, we backcrossed Hdc^rd8/rd8^ mice onto the C57Bl6J background for at least 10 generations. Offspring were genotyped for the deletion of Hdc and the presence of Crb1^rd8/rd8^ and a re-derived Hdc^−/−^ strain free from the *Crb1* mutation was generated. Retinal structural and functional analysis was then performed on cohorts of these Hdc^−/−^ mice that had been raised on a histamine-free diet for 10 days [22].

**Figure 3:**
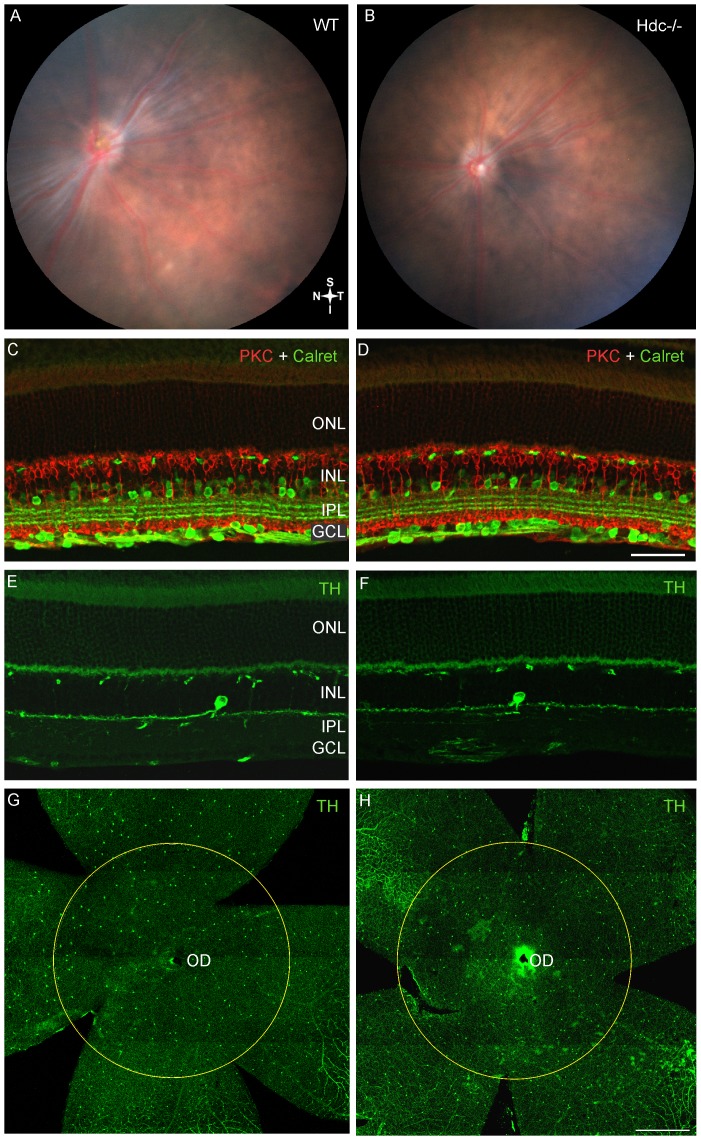
Neuronal integrity of re-derived Hdc^−/−^ mice. Retinal fundus images of a three month old WT (A) and Hdc^−/−^ mouse (B) retina. No lesions were observed in either strain. Vertical sections of WT (C, E) and Hdc^−/−^ mice (D, F) double immunolabeled for the amacrine and ganglion cell marker, calretinin (green; C, D) and the rod bipolar cell marker, protein kinase C (red; C, D), or immunolabeled for the dopaminergic amacrine cell marker, tyrosine hydroxylase, (green; E, F). Retinal wholemounts of WT (G) and Hdc^−/−^ mice (H) immunolabeled for tyrosine hydroxylase (green, G, H). The circles in G, H outline the area of the retina where tyrosine hydroxylase positive amacrine cells were quantified. No gross changes in retinal structure were observed with any neuronal markers examined. Abbreviations as in Fig. 2; Scale bars  = 50 µm (A–D) and 500 µm (E, F).

**Figure 4:**
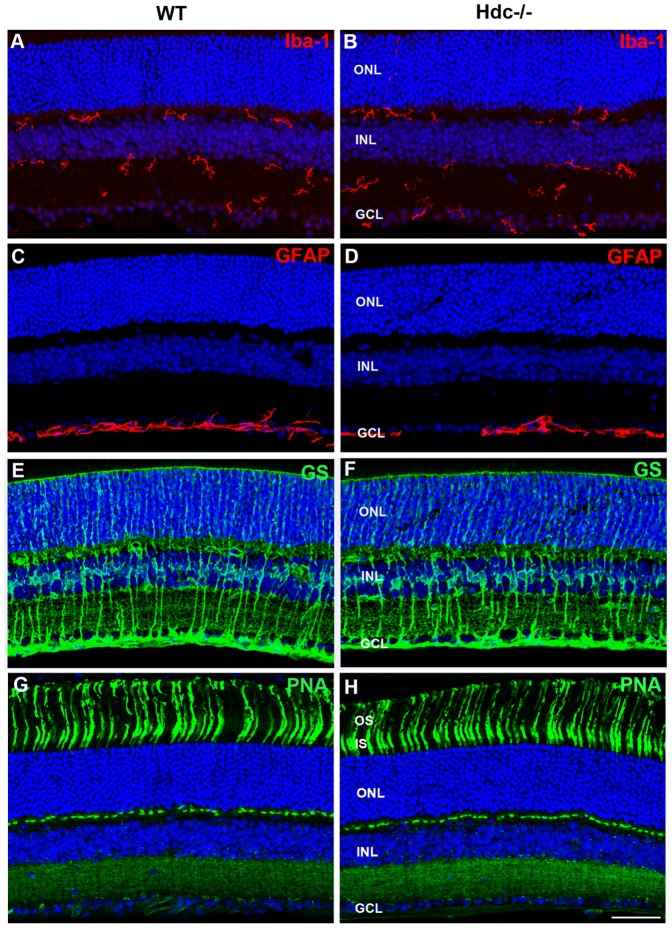
Glial and microglial changes in Hdc^−/−^ mice. Vertical sections of WT- (A, C, E, G) and Hdc^−/−^-mice (B, D, F, H) immunolabeled for the microglial marker, IbA1 (red; A, B), GFAP (red; C, D), glutamine synthetase (green; E, F), and the cone photoreceptor marker, peanut agglutinin (green; G, H). Cell nuclei were labeled with DAPI (blue). There were no apparent increases in microglial number or changes in morphology of microglia in Hdc^−/−^ mice and no gliosis was apparent in the Hdc^−/−^ mouse retina compared to WT mice. Finally, cone photoreceptors appeared no different. Abbreviations as in Fig. 2; Scale bars  = 50 µm.

As shown in Fig. 3, the retinal fundi of adult WT (Fig. 3A) and Hdc^−/−^ mice (Fig. 3B) were indistinguishable, and neither displayed the inferior retinal lesions observed in the original strain (Hdc^rd8/rd8^; Fig. 2B). Immunolabeling for specific retinal cell types showed that all retinal layers were intact (Fig. 3C–F and Fig. 4). Specifically there were no changes in second order, inner retinal neurons such as subclasses of amacrine and ganglion cells (Calretinin, green; Fig. 3C–D; Table 3), and rod bipolar cells (PKC, red; Fig. 3C–D Table 3). Dopaminergic neurons, previously shown to express H_1_R receptors [13], [14] did not appear any different in the two strains of mice (Tyrosine Hydroxylase, TH, green; Fig. 3G–H; Table 3) and showed similar densities in the two strains of mice (Fig. 3G–H; Table 3). As shown in Table 3, although retinal thickness was increased in Hdc^−/−^ retinae, there was no difference in the thickness of the inner plexiform layer, nor in the number of PKCα-IR rod bipolar cells or calretinin immorective cells in the ganglion cells in Hdc^−/−^ compared with wildtype retinae (Table 3). In addition, markers of retinal stress were assessed, including microglial change (IbA–1, red; Fig. 4A–B) and Müller cell gliosis (GS, Green; GFAP, red; Fig. 4C–F). Microglial number and morphology did not appear qualitatively different in Hdc^−/−^ mice (Fig. 4B) compared to WT animals (Fig. 4A). Similarly, Müller cell morphology was similar between the two strains (Fig. 4C–F) and there was no apparent upregulation of GFAP in Müller cells as a result of the deletion of Hdc (Fig. 4C–D). Finally, no differences in cone photoreceptor morphology were noted between the two strains (PNA, green; WT Fig. 4G and Hdc^−/−^ Fig. 4H).

**Table 3:** Mean density of cell types (+SEM) and retinal thickness in wildtype and Hdc^−/−^ mice.

	Control (n = 5–7)	Hdc^−/−^ (n = 6–9)
PKCα (cells/mm)	143+2.1	139+3.0
Calretinin (cells/mm)	536+16.7	579+14.3
Tyrosine hydroxylase (cells/mm^2^)	33+1.4	31+0.8
Total retinal thickness (µm) #	146+2.9	160+3.9
Thickness of Inner Plexiform Layer (µm)	34.9+0.74	36.5+0.96

# denotes statistical significance p<0.05;

**Figure 5:**
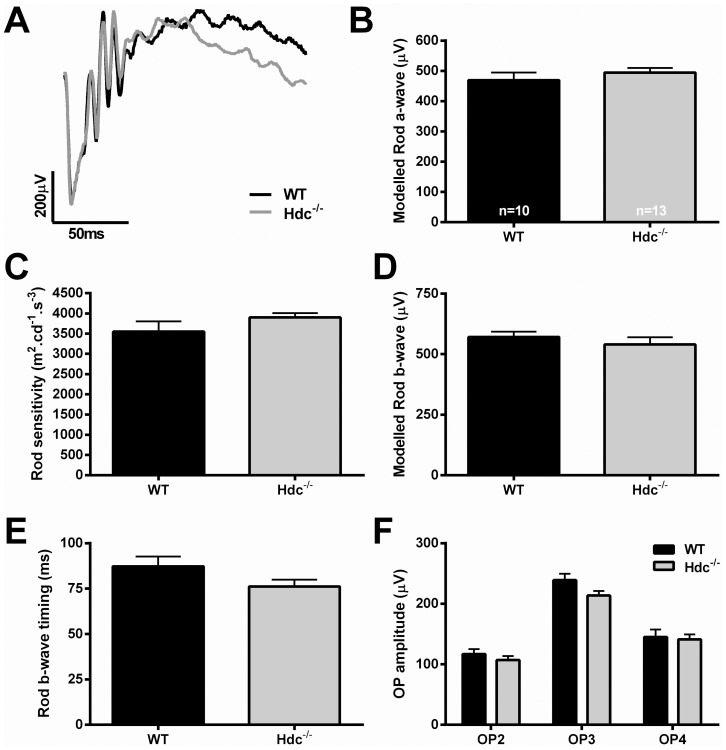
Rod mediated function in Hdc^−/−^ mice. (A) Representative rod mediated ERG waveforms from an adult WT (black) and Hdc^−/−^ mouse (grey). (B, C) Graphs showing the mean +SEM amplitude (B) and sensitivity (C) of the rod photoreceptor response obtained from the modelled rod a-wave amplitude in WT and Hdc^−/−^ mice. (D, E) Graphs showing the mean +SEM amplitude (D) and timing (E) of the post-receptoral response obtained from the modelled rod b-wave. (F) Graph showing the mean +SEM amplitude of the individual rod oscillatory potentials (OP: 2,3 and 4) in WT and Hdc^−/−^ mice. Overall, there were no statistically significant differences between the strains from any of the waveforms examined.

**Figure 6:**
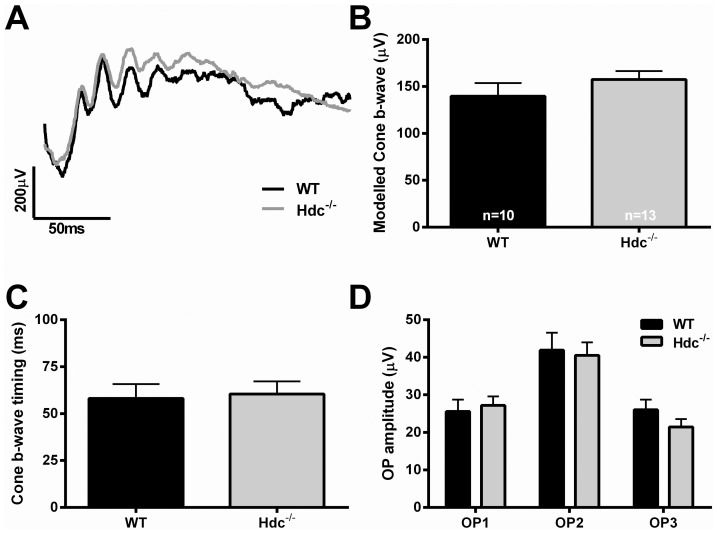
Cone mediated function in Hdc^−/−^ mice. (A) Representative cone mediated waveforms from an adult WT (black) and Hdc^−/−^ mouse (grey). (B,C) Graphs showing the mean +SEM amplitude (B) and sensitivity (C) of the post-receptoral response obtained from the modelled cone b-wave in WT andHdc^−/−^ mice. (D) Graph showing the mean +SEM amplitude of the individual cone oscillatory potentials (OP: 1, 2 and 3) in WT and Hdc^−/−^ mice. Overall, there were no statistically significant differences between the strains from any of the waveforms examined.

Next, we examined whether retinal function was altered in Hdc^−/−^ mice (n = 13) compared to WT controls (n = 10). The ERG is a gross retinal potential that provides information about the function of cohorts of retinal neurons [33]. We used a twin-flash protocol so as to separate rod from cone mediated pathways [31], [32]. Fig. 5 shows representative waveforms of rod mediated function derived from 3 month old WT and Hdc^−/−^ mice (Fig. 5A; WT, black, Hdc^−/−^, grey). Overall, no difference in rod photoreceptor function, either modeled a-wave amplitude (Fig. 5B) or sensitivity (Fig. 5C) was apparent. Similarly, rod mediated inner retinal function, including the modeled b-wave response (Fig. 5D, amplitude; Fig. 5E, time to peak) and the isolated oscillatory potential response (Fig. 5F) was also similar between the two strains. Fig. 6 shows cone mediated function. Owing to the low number of cones in mice, it is only possible to assess cone pathway meditated inner retinal function. Fig. 6A shows representative cone mediated waveforms of WT (A, black) and Hdc^−/−^ mice (A, grey) with the quantification of the modeled b-wave amplitude and implicit time shown in Fig. 6B & C respectively and the cone mediated oscillatory potential responses in Fig. 6D. There were no differences in the amplitude or timing of the cone mediated b-wave response, nor the oscillatory potential responses, in WT compared to Hdc^−/−^ mice. Overall, these results suggest that lack of histamine does not affect the structure and function of the retina in a major way, although, further work is necessary to identify whether specific retinal circuits are affected in the Hdc^−/−^ mice.

### Analysis of blood flow in Hdc^−/−^ mice

**Figure 7:**
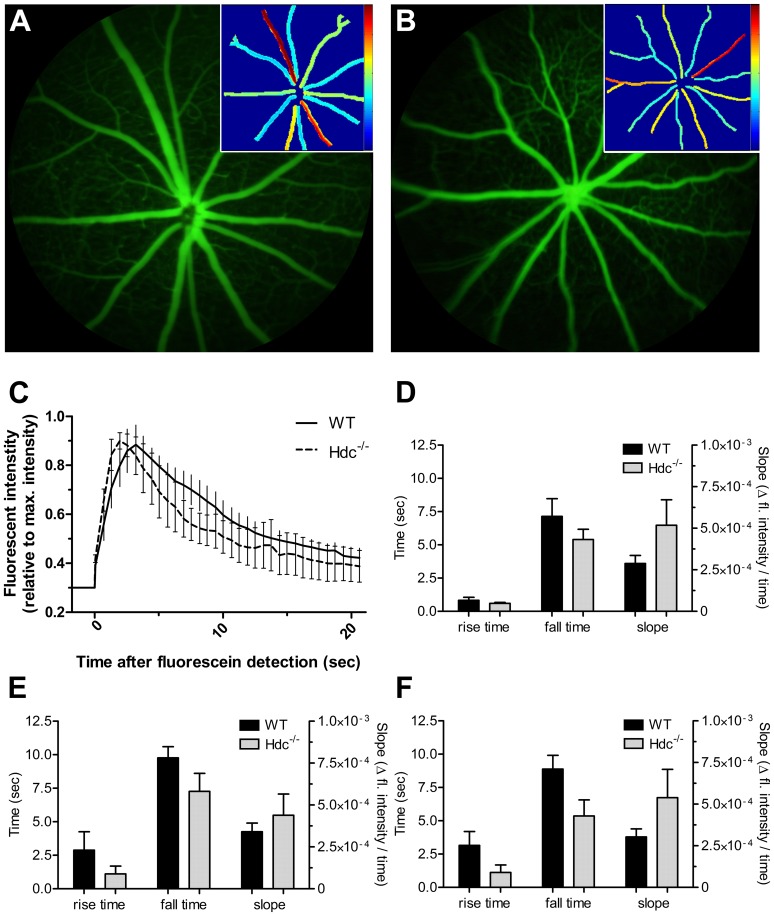
Vascular function in wildtype and Hdc^−/−^ mice. (A, B) Representative fluorescein images from an adult WT(A) and Hdc^−/−^ (B) mouse retina taken at the peak fluorescein intensity. Insets show the fall time determined from pixel-by-pixel analysis of video angiography sequences. (C) graph showing fluorescein dynamics in the major arteries of wildtype and Hdc^−/−^ mice. (D) shows the mean +SEM rise time, fall time and slope for the arteries in wildtype and Hdc^−/−^ whereas those for veins and capillaries are shown in (E) and (F) respectively. Overall, there were no statistically significant differences in any vascular parameter examined.

Histamine has been shown in some studies to alter blood vessel caliber, and potentially blood flow [17], [18]. We assessed blood flow in Hdc^−/−^ (n = 5) and WT mice (n = 5) using video fluorescein angiography along with a pixel-by-pixel analysis to return values for rise time (time to reach 50% brightness), slope (slope of rise) and fall time (time to return to 50% of plateau) within the large blood vessels and the capillaries of the superficial vascular plexus. Figs. 7A and B show representative fluorescein angiograms of a wildtype and Hdc^−/−^ mouse retina at peak fluorescence intensity. A retinal vessel heat map (insets in Fig. 7A, 7B) was used to visualize the time course, where blue is equivalent to a rapid fill (arterioles), while the later fill (venules) is indicated by warmer colours. A representative intensity plot for retinal arteries is shown in Fig. 7C for WT and Hdc^−/−^ animals. The group average for each parameter (rise time, fall time and rise slope) was quantified for retinal arteries (Fig. 7D), the retinal venules (Fig. 7E) and retinal microvasculature (Fig. 7F). There were no apparent differences in the flow of fluorescein through retinal arteries (Fig. 7D), veins (Fig. 7E) or capillaries (Fig. 7F). Similarly, there was no difference in the diameter of arteries or veins (data not shown).

## Discussion

Previous work in the CNS has identified histamine to regulate multiple functions such as sleep-wakefulness, feeding and energy balance, whilst also modulating dopaminergic signaling. There is little data reporting the effect of histamine in the retina. The main findings of this study are that although *Hdc* mRNA is expressed by photoreceptors in the wildtype retina, in Hdc^−/−^ mice lacking the Crb1^rd8/rd8^ mutation, there is no effect on retinal structure, function, nor basal blood flow dynamics. This suggests that in the mammalian retina, histamine only plays a minor role in modulating synaptic signaling and regulating the inner retinal vasculature.

### The original Hdc^−/−^ mouse line harbored the Crb1^rd8/rd8^ mutation

Our results show that the commonly available and reported strain of Hdc^−/−^ mice (subsequently called in this study Hdc^rd8/rd8^) displays a mutation in Crb1 in the background strain. In the retina, Crb1 is expressed by Müller cells [24], and mutations in the gene encoding *Crb1* are associated with severe retinal degeneration [35], [36]. Crb1 has also been described previously in the CNS [37]. Our results examining the retina of the Hdc^rd8/rd8^ are consistent with previous reports on the Crb1^rd8/rd8^ mouse, showing widespread lesions within the inferior temporal retina and rosette formation within the photoreceptor layer [38]. In order to study the role of histamine in the retina, we backcrossed Hdc^rd8/rd8^ mice with C57Bl6J mice to generate an Hdc^−/−^ mouse free of the mutation in Crb1^rd8/rd8^ and then further backcrossed onto the C57Bl6J for at least ten generations.

### Photoreceptors express Hdc but lack of this enzyme has minimal effect on retinal structure and function

The source of histamine in the retina has been the subject of debate for some time, with some authors suggesting that no synthesis of histamine takes place within the mammalian retina, but that histamine containing projections originating from somata within higher brain centers traverse the inner retina [11], [13], [39]. Mast cells, another potential source of histamine, are not present in the retina. Here, we show that *Hdc* mRNA was identified in retinal samples isolated from the outer retina implying that photoreceptors may be a source of histamine synthesis within the retina. It is interesting to note that photoreceptors of invertebrates utilize histamine as their neurotransmitter, and inactivation of *Hdc* in drosophila disrupts the structure of the compound eye [40], [41]. While it is not clear whether *Hdc* mRNA is translated into protein in murine photoreceptors, there is evidence suggesting that histamine may play a role in modulation of retinal function, including localization of histamine receptors [13], [14], [42], and electrophysiological evidence for neuronal modulation in response to histamine, specifically of dopaminergic amacrine cells and neurons in the scotopic pathways [15], [16]. Despite these lines of evidence, our data show no effect of removal of Hdc on global retinal function, as measured by the electroretinogram.

The most likely explanation of these contradictory data is that the modulatory effects of histamine are too subtle to be measured with an ERG. When comparing our findings with previous studies examining the CNS of Hdc^−/−^ mice it is interesting to note that CNS deficits are evident when the availability of histamine is restricted, whereas in the retina this was not observed [10], [20]. Although previous evidence shows that histamine is depleted in these mice following only a few days on a histamine free diet [22], perhaps this is not long enough to produce long lasting effects on retinal function.

Our data shows that Hdc^−/−^ mice display normal retina structure. We found no obvious changes in neuronal number or layering, in microglial response, nor in Müller cell gliosis. Our previous work has shown that even with the subtlest of injuries, microglia and Müller cells respond rapidly [30], [43], [44]. Our data suggest that even though Hdc^−/−^ mice rely on the diet for histamine homeostasis, this is clearly sufficient for preventing any gross morphological changes and activation of glial cells.

Our findings revealed that blood flow dynamics were no different in Hdc^−/−^ compared to wildtype mice. Previously, studies have suggested that histamine vasodilates ocular blood vessels, via actions on endothelial cells [18], [45]. Moreover, intravenously administered histamine has been shown to cause an increase in mean blood flow in the ophthalmic artery as well as in blood vessels of the choroid [17], [18], [46]. In contrast, one study showed that histamine had no effect on red blood cell velocity, nor retinal blood flow [17], [18]. Our result implies that either histamine plays only a very minor role in modulation of the blood flow in the retina, or that the net systemic effect of lack of histamine in the Hdc^−/−^ mice results in retinal blood flow being normal. Whether the absence of histamine modifies the capacity of the retinal blood vessels to autoregulate requires further investigation. In addition, further work is necessary to determine whether histamine affects blood flow in the mouse retina in an acute manner.

In conclusion, this study examining the retinal structure and function of the Hdc^−/−^ mouse showed that lack of histidine decarboxylase, the rate limiting enzyme for histamine formation, has little effect on retinal structure or function. More work is necessary, however, to gain a better understanding of why Hdc is present in the outer retina and whether histamine plays a role in modifying specific retinal circuits.

## Supporting Information

S1 Fig Histamine labelling is absent in Hdc^−/−^ mice. Transverse sections of the brain through the tuberomammillary nucleus of the hypothalamus from (A) a C57Bl6J wildtype mouse raised on a conventional diet, (B) an Hdc^−/−^ mouse raised on a a histamine free diet. Histamine-immunoreactive somata were detected in the tuberomammillary nucleus of the WT nucleus, but not in tuberomammillary nucleus from the Hdc^−/−^-mice. Scale bars = 200 µm.
(TIF)
