# Supplementary figures and images for: The Role of Histamine in the Retina: Studies on the Hdc Knockout Mouse
Source: PLoS One. 2014 Dec 29;9(12):e116025. doi: 10.1371/journal.pone.0116025 (PMC4278841; doi:10.1371/journal.pone.0116025)

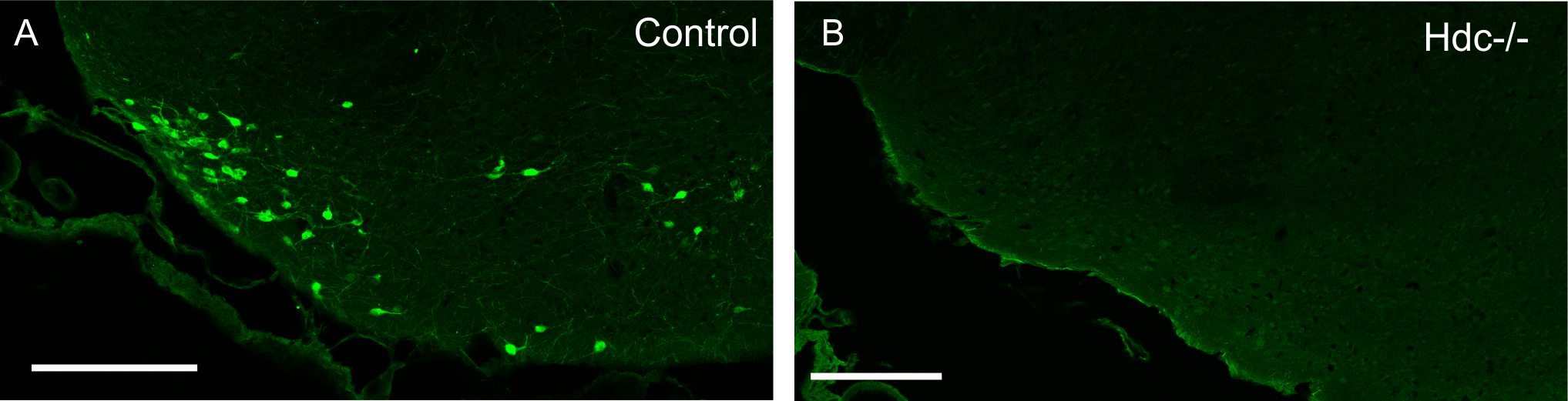

Supplement: S1 Fig — Histamine labelling is absent in Hdc−/− mice. Transverse sections of the brain through the tuberomammillary nucleus of the hypothalamus from (A) a C57Bl6J wildtype mouse raised on a conventional diet, (B) an Hdc−/− mouse raised on a a histamine free diet. Histamine-immunoreactive somata were detected in the tuberomammillary nucleus of the WT nucleus, but not in tuberomammillary nucleus from the Hdc−/−-mice. Scale bars = 200 µm. (TIF) [file pone.0116025.s001.tif]
